# Great spotted cuckoo nestlings have no antipredatory effect on magpie or carrion crow host nests in southern Spain

**DOI:** 10.1371/journal.pone.0173080

**Published:** 2017-04-19

**Authors:** Manuel Soler, Liesbeth de Neve, María Roldán, Tomás Pérez-Contreras, Juan José Soler

**Affiliations:** 1 Departamento de Zoología, Facultad de Ciencias, Universidad de Granada, Granada, Spain; 2 Dep. Biology, Terrestrial Ecology Unit, Ghent University, Gent, Belgium; 3 Departamento de Ecología Funcional y Evolutiva, Estación Experimental de Zonas Áridas (CSIC), Almería, Spain; Consiglio Nazionale delle Ricerche, ITALY

## Abstract

Host defences against cuckoo parasitism and cuckoo trickeries to overcome them are a classic example of antagonistic coevolution. Recently it has been reported that this relationship may turn to be mutualistic in the case of the carrion crow (*Corvus corone*) and its brood parasite, the great spotted cuckoo (*Clamator glandarius*), given that experimentally and naturally parasitized nests were depredated at a lower rate than non-parasitized nests. This result was interpreted as a consequence of the antipredatory properties of a fetid cloacal secretion produced by cuckoo nestlings, which presumably deters predators from parasitized host nests. This potential defensive mechanism would therefore explain the detected higher fledgling success of parasitized nests during breeding seasons with high predation risk. Here, in a different study population, we explored the expected benefits in terms of reduced nest predation in naturally and experimentally parasitized nests of two different host species, carrion crows and magpies (*Pica pica*). During the incubation phase non-parasitized nests were depredated more frequently than parasitized nests. However, during the nestling phase, parasitized nests were not depredated at a lower rate than non-parasitized nests, neither in magpie nor in carrion crow nests, and experimental translocation of great spotted cuckoo hatchlings did not reveal causal effects between parasitism state and predation rate of host nests. Therefore, our results do not fit expectations and, thus, do not support the fascinating possibility that great spotted cuckoo nestlings could have an antipredatory effect for host nestlings, at least in our study area. We also discuss different possibilities that may conciliate these with previous results, but also several alternative explanations, including the lack of generalizability of the previously documented mutualistic association.

## Introduction

Coevolutionary interactions are very common and widespread in nature [1]. They are frequently complex and sometimes mutualism and parasitism can exist in the same system depending on the interacting species and ecological context [1,2]. Some interactions are however considered purely antagonistic, such as those involving avian brood parasites and their hosts, in which hosts are forced to rear completely unrelated chicks at the cost of losing their own offspring [3,4].

The great spotted cuckoo (*Clamator glandarius*) is a non-evictor brood parasite that lays its eggs in the nests of magpies (*Pica pica*, primary host) and carrion crows (*Corvus corone*, secondary host) [5]. Recently, Canestrari et al. [6] found in their study area that great spotted cuckoos benefit their carrion crow host in contexts of high predation risk. Cuckoo chicks produce a malodorous cloacal secretion when they are grabbed, which apparently deters predators from parasitized host nests. Presumably, this antipredatory effect would result into more fledglings in parasitized nests compared to non-parasitized nests during breeding seasons with high predation risk. The experimental repellence tests which showed that the malodorous cloacal secretion discouraged potential nest predators (mammals, corvids, and raptors) from consuming food with cloacal-secretion added, as well as the association between experimental parasitism and predation rate reported in the paper, support the expected benefits, which would overcome costs of brood parasitism at high predation rates in the population [6].

Thus, the outcome of host-parasite interactions in the great spotted cuckoo—carrion crow system would fluctuate yearly between parasitism and mutualism depending on the intensity of predation pressure [6]. Quite some time ago, Smith [7] also reported an unusual interaction between giant cowbirds (*Scaphidura oryzivora*) and their hosts. Botflies (*Philornis* spp.) infest both parasite and host nestlings and can cause nestling mortality. If host nests were located near colonies of aggressive wasps, which prevent botflies from parasitizing nestlings, hosts rejected non-mimetic eggs; but in absence of wasps they accepted non-mimetic eggs because presumably giant cowbird nestlings removed subcutaneous botfly larvae from themselves and from host nestlings [7]. Then, in this presumed case of preening mutualism, giant cowbirds benefit from a tolerance by the hosts, and hosts benefit because parasitic nestlings remove botfly larvae from their nestlings. However, subsequent studies found that hosts behave aggressively against giant cowbirds and reject most non-mimetic eggs regardless of the presence of protecting wasps [8,9] and so did not find confirming evidence for preening mutualism [9,10].

In order to evaluate whether the results found by Canestrari et al. [6] in northern Spain with the carrion crow can be replicated in other populations and other host species, we here compared predation rates of naturally and experimentally parasitized and non-parasitized nests of both the carrion crow and the magpie in a population located in southern Spain. The magpie is slightly larger than the great spotted cuckoo while the carrion crow is more than twice the weight of the brood parasite [5]. Magpies respond aggressively towards adult great spotted cuckoos and they are able to reject foreign eggs laid in their nests [11,12], while carrion crows lack these defensive mechanisms [5,13]. In our study population (contrary to the population studied by Canestrari et al. [6]), magpies are the preferred hosts [5,11,14], while carrion crows are mainly used at the beginning of the cuckoo breeding season when magpie nests are scarce [5]. Because of the superior competitive ability of carrion crow nestlings (i.e. larger size), it is more costly for cuckoos to parasitize carrion crow nests compared to those of the magpie [5]. Magpies are therefore the species suffering the highest cost of parasitism [5,15,16], probably explaining the evolution of defences in this host species and not in the carrion crow [5].

Parasitized magpie nests fledge on average 0.7 of their own young (compared to 3.6 own young in unparasitized nests) while parasitized nests of carrion crows fledge on average 1.6 of their own young (compared to 3.1 in unparasitized nests) [5]. These host nestlings starve due to competition with cuckoo nestlings usually during the first days of the nestling period. Therefore, any possible anti-predator effect by cuckoo nestlings after this point is less beneficial for magpies than for carrion crows. However, it can be expected that any effect of cuckoo defences on predators would still be detectable in magpies. Thus, looking for an effect in magpies is clearly of interest.

Here, we test the hypothesis of an association between parasitism and predation rate by analysing information recorded in both magpie and carrion crow host nests, and by using different experimental scenarios in which parasitic hatchlings were added or removed in a subset of nests of both host species.

## Material and methods

### Ethics statement

Research has been conducted according to relevant Spanish national (R. D. 1201/2005, de 10 de Octubre) and regional guidelines. All necessary permits, including those concerning cross-fostering manipulations, were obtained from the Consejería de Medio Ambiente de la Junta de Andalucía, Spain. Approval for this study was not required according to Spanish law because it is not a laboratory study in which experimental animals have to be surgically manipulated and/or euthanatized. Our study area is not protected but privately owned, and the owners allowed us to work in their properties. This study did not involve endangered or protected species. The great spotted cuckoo is included in both Spanish national (R. D. 139/2011, 4 February) and regional (D. 23/2012, 14 February) lists of species under special protection, but not in the catalog of endangered species of any of the two entities, although it is protected according to the Bern Convention, Appendix II.

Cross-fostering manipulations were made by carefully transporting the nestlings in an artificial cotton nest lined with tissues, maintaining the temperature in the car between 25–30°C. Cross-fostering per se does not affect nestlings or host parents’ behaviour [17–19]. In some cases in which we took one great spotted cuckoo chick that was alone in the nest, we left another cuckoo chick of similar age from a multiparasitized nest to avoid nest abandonment.

### Study area and host populations

The Hoya de Guadix (Southern Spain; 37° 18 N, 3° 11′ W) is a high-altitude plateau (approx. 1000 m a.s.l.) with extensive non-cultivated areas, cereal crops (especially barley), almond (*Prunus dulcis*) orchards, and some holm-oak trees (*Quercus rotundifolia*). More detailed information on the study area can be found elsewhere [11,20]. Potential nest predators in the area are stone martens (*Martes foina*), genets (*Genetta genetta*), jackdaws (*Corvus monedula*), common ravens (*Corvus corax*), magpies and carrion crows.

The Guadix magpie population is composed of several nearby plots that differ significantly in ecological conditions including food availability, magpie density or brood-parasitism rates [21–23]. Brood parasitism by the great spotted cuckoo is very common in magpie nests within the study area (54.8%, *n* = 778 [23]).

Carrion crows are present only in some plots, mainly in those with holm-oak trees. This area is located at the southern limit of the distribution of the species [24]. In our study area crows breed cooperatively; on average, 66% of the breeding attempts in the study area presented cooperative breeding and the average group size was 3.0 (range from 2 to 7; [25]). Group size has not been measured in this study, and therefore could not be controlled for statistically. Crows are parasitized at a lower rate than magpies (28.5%, *n* = 144 [5]).

Data presented in the Results section or Supplementary material were never published before.

### General field procedures

In each breeding season, we started to search for nests about two weeks before the start of egg-laying (around mid and end of March, for carrion crows and magpies, respectively). After a nest was located, it was visited at least two times per week in the case of magpies or every four days in the case of carrion crows, but at the predicted hatching date, nests were visited daily. This frequent monitoring enabled us to determine laying date, clutch size, occurrence of brood parasitism and number of parasitic eggs, hatching success, and number of fledged young in each nest. A nest was considered to have been depredated when it was found empty before the predicted date of fledging. Misclassification of predation in parasitized and unparasitized nests is highly unlikely given that depredated nests are easily recognizable, because either the dome was destroyed or the lining nest material was completely messed up. We also recorded the number of dead or missing nestlings, which we assumed to be due to starvation when they were the smallest/youngest nestlings in the nest (nestlings were individually marked at hatching with a non-toxic marker on their tarsus and this mark was repeated in each nest visit). In our study area, partial predations only rarely occur and always eventually result in the complete depredation of the nest ([25], personal observation in the present study). Overall, we collected complete information (clutch size, laying date, brood parasitism, hatching and fledging success) for 736 magpie nests over eight years (2006–2013), and for 120 carrion crow nests over four years (2006–2009).

### Translocation experiment

Between 2007 and 2009 we created experimental broods in magpies by means of translocation of recently hatched nestlings with the purpose of testing several hypotheses related to food delivery by parents and growth rate of nestlings according to the nest content (see [18]). Hatchlings (0–1 days old) were cross-fostered by carefully transporting them by car in an artificial cotton nest with temperature between 25 and 30°C. We manipulated broods soon after hatching to obtain cuckoo and magpie/crow nestlings of similar age within each nest, thereby aiming to ensure their survival until fledging. Detailed information about the experimental procedures can be found in Soler and de Neve [18]. In short, we manipulated parasitism state and created the following experimental nests: “cuckoo removed” (cuckoo hatchling(s) were removed from naturally parasitized nests and replaced by magpie hatchlings); “cuckoo added” (cuckoo hatchling(s) were added to naturally non-parasitized nests); and two groups of unmanipulated nests “control parasitized” and “control non-parasitized”. Although great spotted cuckoos have been shown to retaliate upon magpies that eject parasitic eggs (mafia behaviour; [26]), we did not observe any evidence of experimental magpie nests in which the cuckoo hatchlings were removed and replaced by magpie nestlings to be depredated by great spotted cuckoos. This is not surprising because first, mafia behaviour is expected to occur at the egg stage when the cuckoo egg is ejected [26], and second, mafia behaviour and magpies’ response to retaliation by great spotted cuckoos vary greatly among areas depending on ecological conditions [27].

In carrion crows, the translocation experiment was not performed in parasitized nests due to low sample sizes (i.e. in none of the parasitized nests the cuckoo chick was experimentally removed). However, during the course of the study (2006–2009), parasitism status of non-parasitized crow nests was manipulated in 14 nests by adding one cuckoo nestling (1–2 days old) soon after hatching of the first crow nestlings (0–1 days old; 0 is the day on which hatching occurs). Brood size did not significantly differ between control nests (either parasitized or non-parasitized) and “cuckoo added” nests (F_2,77_ = 1.03, *p* = 0.36). As we followed the fate of experimental nests of magpies and carrion crows until fledging, this experimental setup allowed us to examine a causal relationship between nest content and nest predation. Great spotted cuckoos, at least in magpie nests, did not develop worse in nests with same-aged host nest-mates compared to when alone or together with other cuckoos in the nest [17]; thus the production of the fetid secretion should not have been affected.

### Statistical analyses

To analyse variation in predation and parasitism rate between years, Generalized Linear Models (GLZ) were used with binomial error distribution and logit link function.

Variation in predation rate in relation to parasitism state was analysed with Generalized Linear Mixed Models (GLMM) with binomial error distribution and logit link function in which year, plot identity, and the interaction between year and plot identity were included as random terms in order to statistically control for variation in dependent and independent factors that was not directly related to the hypothesis tested. In models built to explain variation in predation rate during the egg-phase, laying date was included as a covariate, while in models focusing on the nestling phase brood size was included as an additional covariate in order to account for a possible influence of these variables on predation risk.

The translocation experiment (i.e. manipulation of parasitism state during the nestling period) was mainly undertaken in magpie nests during the years 2007–2009, and so analyses (GLMM) were based on the subset of nests from those years. Experimental treatment (control parasitized versus cuckoo removed or control non-parasitized versus cuckoo added) was included as a fixed explanatory factor, and brood size and hatching date as covariates.

Odds ratios of predation rates of parasitized and unparasitized nests during the egg and nestling stages, as well as 95% CI were estimated following Altman [28] as implemented in MEDCAL (https://www.medcalc.org/calc/odds_ratio.php). The association between annual predation rates and odds ratios was explored by means of a GLM with odd ratios as dependent variables, reproductive phase as independent factor and annual predation as continuous predictor. Whether the association between annual predation and odds ratios differed depending on reproductive cycle was explored by the interaction term that was estimated in a separate model that also included the main effects. These models were weighted by the number of nests checked during each of the study years.

All analyses were performed with SAS 9.4 (SAS Institute Inc., Cary, NC, USA) and figures were made with STATISTICA 7.0 (StatSoft Inc. 2004, Tulsa, USA). Given the binomial error distribution in all analyses, effect sizes are given on a logit scale as mean differences between groups (± SE).

## Results

**Fig 1:**
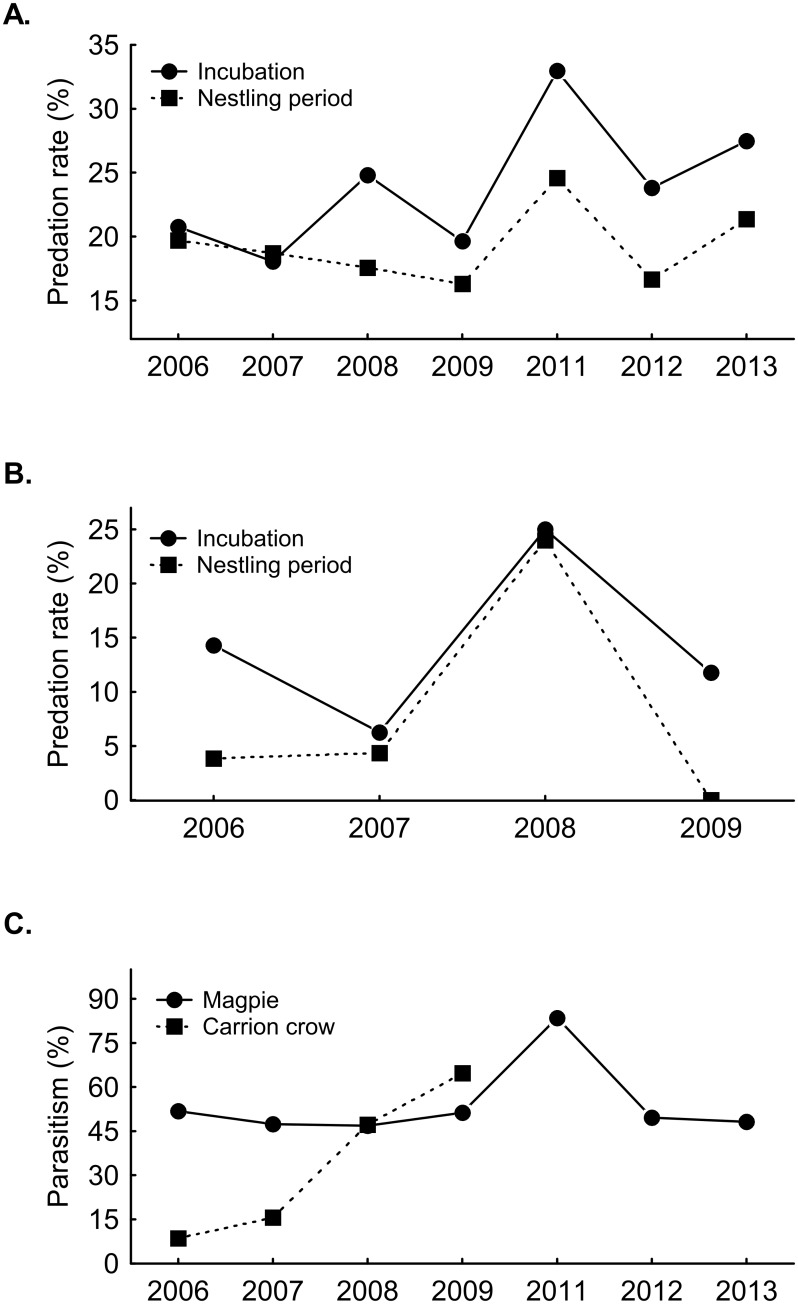
The probability of nest predation in magpies (panel A) and carrion crows (panel B) during the incubation and nestling phase, and nest parasitism (panel C) in magpies and carrion crows in the different study years. Table 2 shows the outcome of the models.

During the incubation period, predation rate in magpie and carrion crow nests did not vary significantly between years (Table 1; Fig 1). During the nestling period, predation rate did not vary between years in magpies, but it did in crows (Table 1; Fig 1).

**Table 1:** Results of Generalized Linear Models exploring variation between study years in predation rate and parasitism rate during incubation and during the nestling period, in magpie and crow nests.

	*χ* ^2^	df	p	N
**Magpie**				
**Predation**				
Incubation	6.59	6	0.36	736
Nestlings	6.94	6	0.32	598
**Parasitism**	50.37	6	<0.001	736
**Crow**				
**Predation**				
Incubation	4.97	3	0.17	120
Nestlings	7.75	3	0.02	80
**Parasitism**	26.5	3	<0.001	120

Parasitism varied between years in both crow nests and magpie nests (Table 1). In crow nests parasitism strongly increased during the course of the years (Fig 1C), while in magpies there was one year with an exceptional high rate of parasitism (83.5%), while in the remaining study years there was little variation in parasitism rate (range 47–52%, Fig 1C).

**Fig 2:**
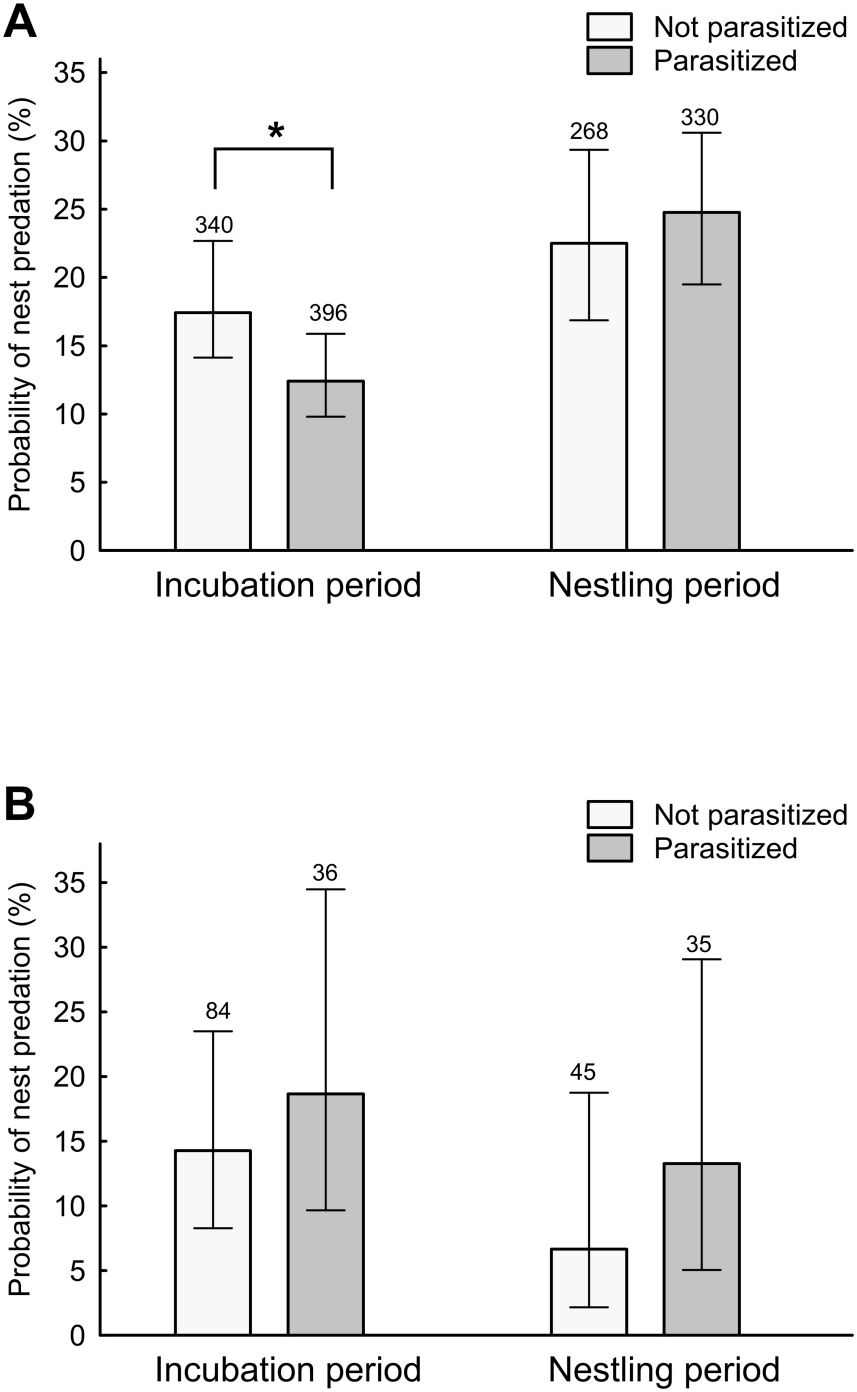
Proportion of parasitized and non-parasitized nests of magpies (panel A) and carrion crows (panel B) that were depredated during the incubation and nestling period. Sample sizes are depicted above bars. Vertical lines represent 95%CI.

During incubation, non-parasitized magpie nests were more frequently depredated compared to parasitized magpie nests, while during the nestling stage (including all monitored nests) this was not the case (Table 2, S1 Table, Fig 2a; covariate brood size F_1,569_ = 1.04, *p* = 0.31). In carrion crow nests, there was no significant difference in predation rate between parasitized and non-parasitized crow nests during both incubation and nestling phase (Table 2, S2 Table, Fig 2b; covariate brood size: F_1,47_ = 2.33, *p* = 0.13).

**Table 2:** Results of Generalized Linear Mixed Models testing differences in predation rate between non-parasitized versus parasitized magpie and crow nests.

	*Estimate ± SE*	df	F	P
**Magpie**				
Incubation	4.65 ± 2.04	1,707	4.94	0.026
Nestlings	-2.25 ± 2.24	1,569	0.28	0.60
**Crow**				
Incubation	4.55 ± 5.64	1,83	0.21	0.65
Nestlings	6.82 ± 7.78	1,47	0.59	0.45

**Fig 3:**
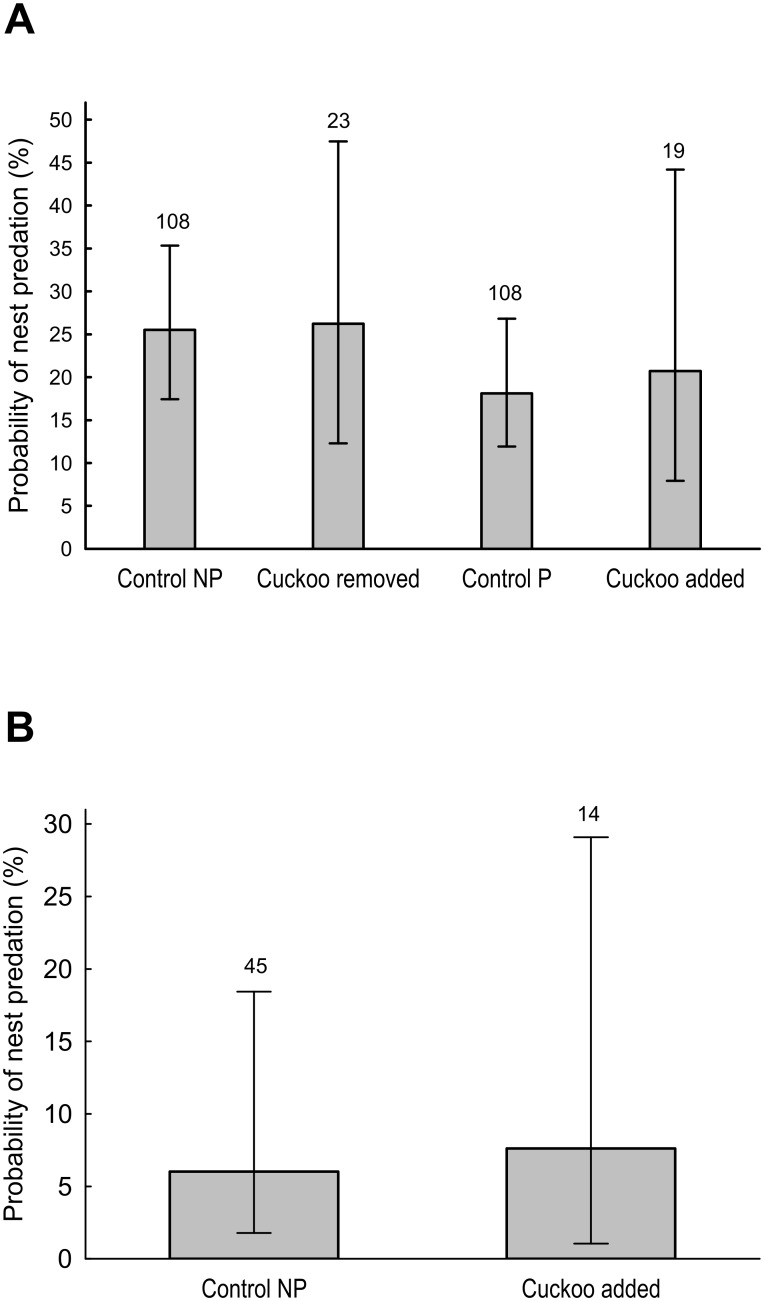
Probability of predation in control (non-parasitized = NP, parasitized = P) and experimental nests (cuckoo removed, cuckoo added) of magpies (panel A) and carrion crows (panel B) during the nestling period. Numbers above bars represent sample size. Vertical lines represent 95%CI.

The translocation experiment of great spotted cuckoo hatchlings during 2007–2009 did not reveal causal effects between parasitism state and predation rate of magpie nests. Among initially parasitized nests, those from which cuckoo hatchlings were experimentally removed did not suffer higher predation rates compared to control parasitized nests (GLMM, F_1,112_ = 0.74, 8.24 ± 9.10, *p* = 0.39, Fig 3a). The experimental parasitism of magpie nests that were not selected for parasitism by cuckoos did not reduce the probability of predation (GLMM, F_1,114_ = 0.05, -4.21 ± 6.38, *p* = 0.82, Fig 3a). In fact, pairwise comparisons between all four-treatment groups resulted non-significant (all *p* > 0.39).

In carrion crow nests, the translocation experiment did not affect predation rate, which was similar between control non-parasitized nests and non-parasitized nests where a cuckoo hatchling was added experimentally (GLMM, F_1,54_ = 0.01, -0.7 ± 12.18, *p* = 0.95, Fig 3b).

**Fig 4:**
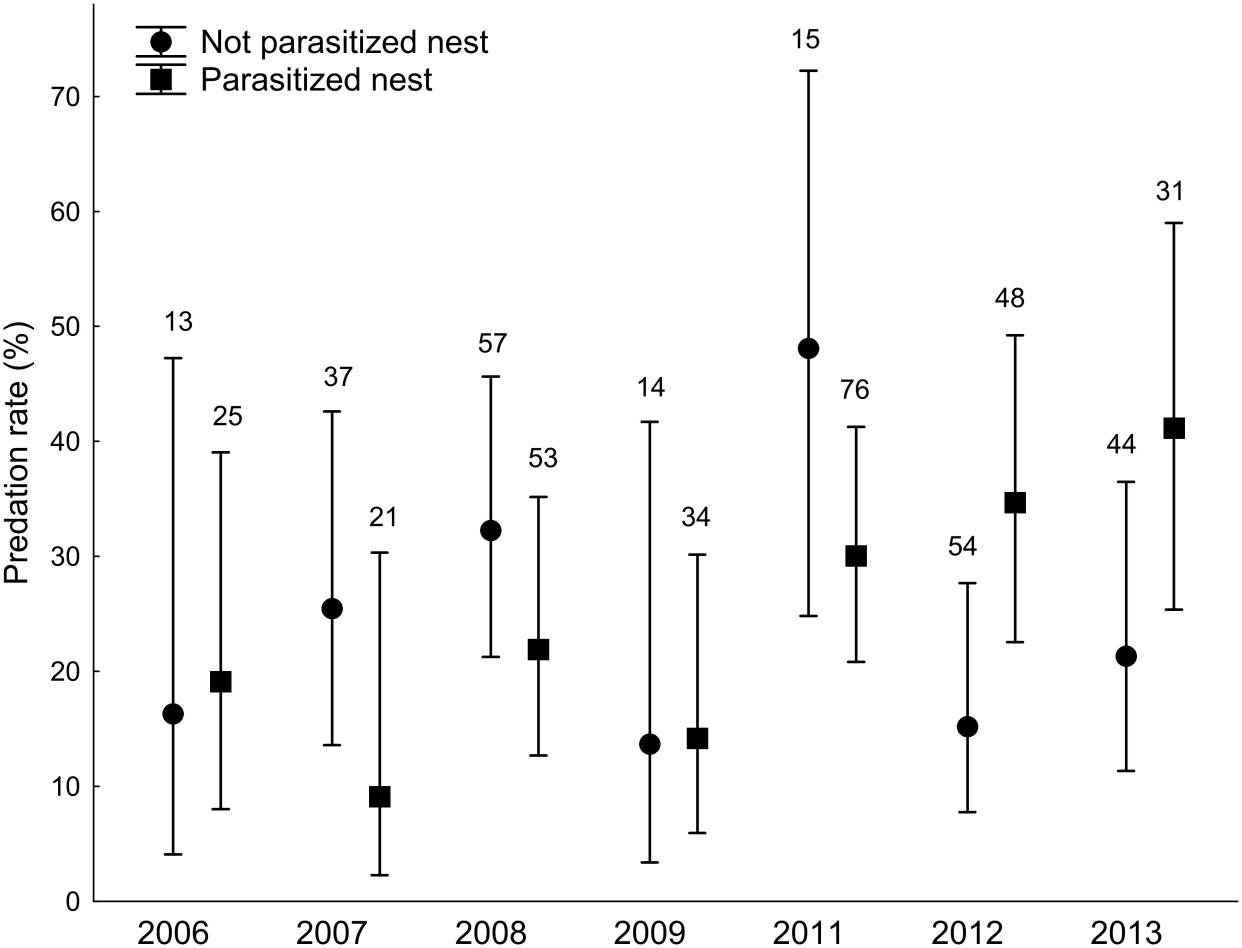
Interaction between year and parasitism state on the probability of predation during the nestling period in magpie nests. Bars represent 95%CI.

**Fig 5:**
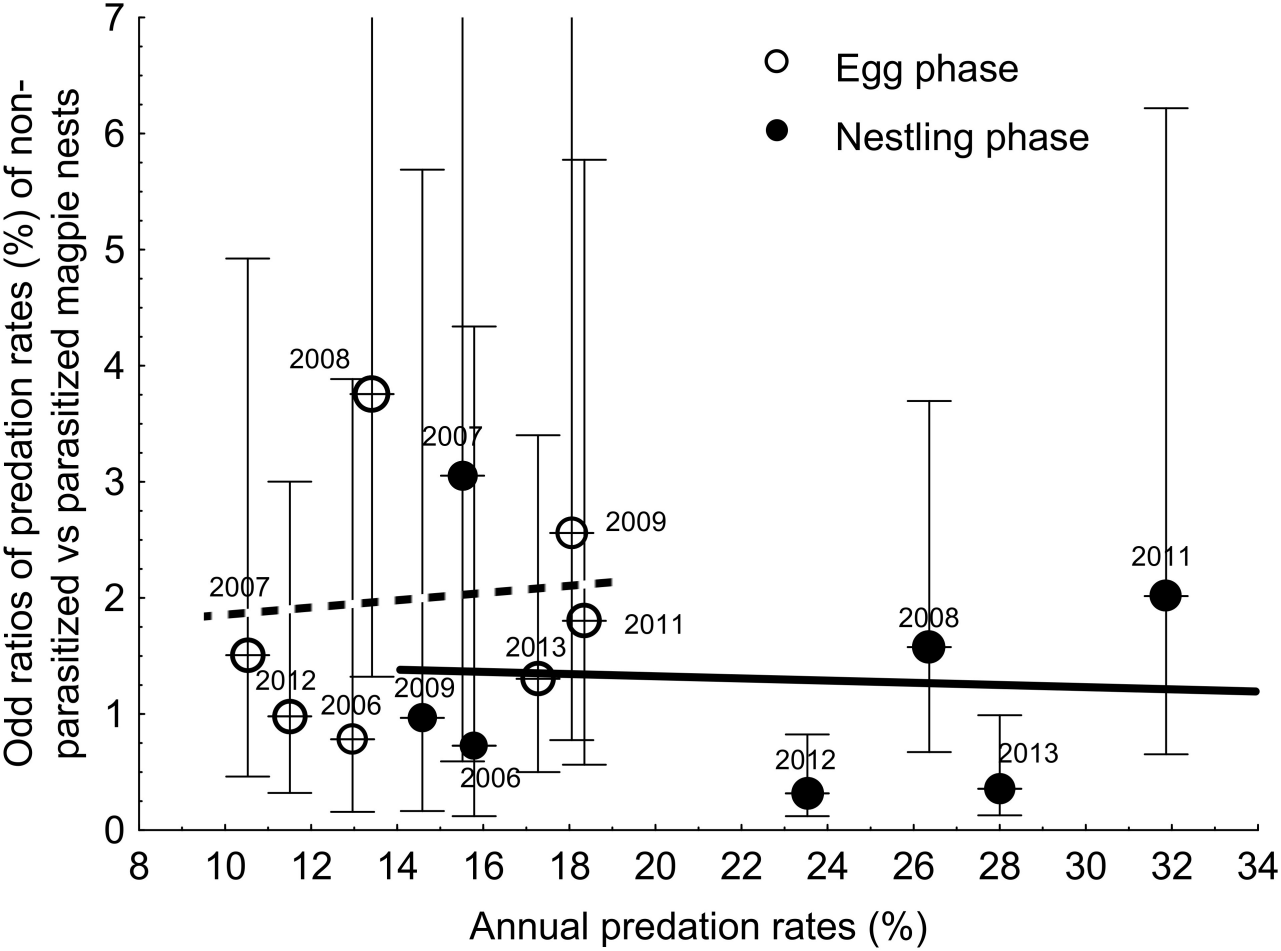
Relationships between annual predation rates and annual odd ratios of predation rates of non-parasitized and parasitized magpie nests (only non-manipulated nests considered) during the egg (open circles and dashed line) and nestling (solid circles and solid line) phases. Lines are weighed regression lines and point sizes are proportional to log-transformed number of nests per year. 95% CI of odd rations are also shown. Scaling of the y-axe do not show the upper CI value for odds ratios values of 2008 (upper CI = 10.7) and 2009 (upper CI = 8.5) for the eggs stage, and of 2007 (upper CI = 15.7) for nestlings phase.

For magpie predation rates during the nestling stage, there exists an apparent discrepancy between Figs 2 and 3 given that Fig 2a shows, for parasitized nests, a pattern to be more depredated than non-parasizited nests while this pattern is opposite in Fig 3a. This apparent discrepancy is provoked by a significant interaction between the state of parasitism and year on the observed predation rate in magpies. Predation rate of parasitized magpie nests during incubation was lower than during the nestling period, but during the nestling period there was a significant interaction with year (see Fig 4). During 2006 and 2012–2013, parasitized nests were more depredated than non-parasitized nests, while during the other breeding seasons there was the opposite trend, which could be due to a change in the predator community (for instance, when a stone marten or a genet is present in a concrete area, most of the magpie nests in that area are depredated). Odds ratios in predation rates did not differ for nestling and egg phases (GLM weighed by sample size, F = 0.67, df = 1,11, *p* = 0.43) and did not associate with annual predation rates (GLM weighed by sample size, F = 0.01, df = 1,11, *p* = 0.991, Fig 5). Furthermore, the association between differences in predation rates of parasitized and non-parasitized nests and annual predation rates was similar for egg and nestling phases (GLM weighed by sample size, interaction between phase and annual predation rates: F = 0.07, df = 1,10, *p* = 0.800).

## Discussion

In parasitized nests, the risk of predation usually increases from the egg to the nestling stage [15,29]. This is probably the consequence of the typical increased intensity and loudness of the begging calls emanating from parasitized nests, which can directly attract predators [30,31]. A causal effect of the presence of brood parasites on nest predation risk has been demonstrated in the blackbird (*Turdus merula*), where experimental parasitism with great spotted cuckoo nestlings doubled the probability of predation [32]. Great spotted cuckoo nestlings, as brood-parasitic nestlings in general, also display exaggerated begging behaviour and display much longer calls compared to magpie nestlings [33,34]. However, nestlings from this cuckoo species also void a fetid cloacal secretion when grabbed in the nest. The cloacal secretion of great spotted cuckoo nestlings has a foul smelling, and a causal link of its repellent function for carrion crow nest predators has been demonstrated [6].

During the incubation phase non-parasitized magpie nests were depredated more frequently than parasitized nests, which could be a consequence of great spotted cuckoos selecting nests with a lower probability of becoming depredated, as has recently been demonstrated experimentally [29]. However, contrary to the predicted lower predation rate of parasitized nests during the nestling phase, our analyses of the long-term data set showed that parasitized nests were not less frequently depredated compared to non-parasitized ones in either host species. Our experimental data could not demonstrate any causal relationship between parasitism state and nest predation risk (Fig 3a and 3b).

Our results, and previously published information, do not completely guarantee that the fetid cloacal secretion from cuckoo chicks is the reason for the detected lower predation rate of parasitized nests found in another carrion crow population [6]. First, this secretion did not protect parasitized blackbird nests in the experimental study mentioned above [32]; and second, even if predators may avoid cuckoo nestlings, they could still depredate on host nestlings. Great spotted cuckoo nestlings do not excrete the malodorous secretion when they are scared in the nest (personal observation), as does happen with hoopoe (*Upupa epops*) nestlings that are capable of throwing their faeces and cloacal secretions directionally against the predator [35], but only when they are grabbed (personal observation). As indirect evidence supporting the unpalatability of cuckoos in natural conditions, Canestrari et al. [6] reported that two out of 13 cuckoo chicks presented injuries just before fledging, presumably caused by a predator that released the chick after grasping it. The possibility of intra-nest aggression was discarded because in 300 hours of video-recordings at parasitized nests such behaviour was never observed. However, direct evidence of a predator releasing a cuckoo chick is also lacking today. During nearly 30 years of studying great spotted cuckoos, we have handled hundreds of cuckoo chicks just before fledging [36–38], but we have never found any injured cuckoo fledgling in either magpie or crow nests. Thus, the protection of crow nestlings would occur only in the case that predators attack the cuckoo chick first, which presumably would happen only in a fraction of predation attempts. In any case, protection of the entire brood is unlikely. Therefore, we consider that malodours secretions should be conservatively considered as a cuckoo self-protection mechanism. In fact, some other cuckoo species that do not share the nest with host nestlings (i.e. evicting cuckoos), as the common cuckoo (*Cuculus canorus*), also release defensive secretion [39–41].

An alternative explanation for the detected reduced probability of predation associated to experimental parasitism detected by Canestrari et al. [6] is related to possible side effects of the experimental protocol. Donor and receiver nests of parasitic hatchlings were synchronized in relation to laying date (not hatching date) of carrion crow eggs. Given that great spotted cuckoos on average hatch 6–7 days earlier than their carrion crow hosts [5], by transferring cuckoo hatchlings into non-parasitized nests synchronized by laying date, the experiment simulated the predation of the only nestling present in the nest for donor adult hosts, and an advance of hatching time for receiver adults; factors that could affect future probability of predation. On the one hand, partial nest predation provokes a reduction in nest defence [42] and an increase in future predation risk [43]. On the other hand, parents that received a cuckoo nestling several days before the expected hatching date could increase risk taking and investment in nest defence given that the presence of early hatchlings, early feathered nestlings (great spotted cuckoos start to develop feathers few days after hatching [44]) and nestlings begging for food vigorously as great spotted cuckoos do [33,34], would indicate that they have one or two nestlings considered of good phenotypic quality [45]. Although it can be argued that Canestrari et al. [6] did not detect differences in predation rate between experimental and control treatments, knowledge on baseline effects of early hatching and of partial predation on the probability of predation in carrion crow nests is indispensable to evaluate net effects of translocation experiments, even more when considering a relative low sample size (i.e., statistical power). Our experimental treatment in magpies and carrion crows ruled out the most important confounding aspect (i.e. partial nest predation and sudden appearance of an early hatchling) given that we never left a nest without nestlings when performing translocation experiments and cuckoo hatchlings were introduced in magpie and crow nests when at least one nestling had hatched [5]. A similar experimental approach in areas of high predation rates of carrion crow nests is therefore necessary to reach firm conclusions.

With respect to the exploration of a correlation between differences in fledgling production between parasitized and non-parasitized nests and annual predation rate [6], in magpies we analyzed whether differences in the probability of predation among parasitized and non-parasitized magpie nests covaried with annual predation rates in non-manipulated nests. We analyzed separately the egg and nestling phases. Overall, we did not find evidence supporting the expected differential antipredatory effect of parasitism in years of high predation rates. Differences (odds ratios) in predation rates between parasitized and non-parasitized nests were similar for nestling and egg phases and did not associate with annual predation rates in neither of the breeding stages (Fig 5). Thus although also for magpies the number of years with appropriate information is limited, no evidence of differential antipredatory effects of great spotted cuckoos during years of elevated predation rates was detected.

The significant correlation found in the study of Canestrari et al. [6] is based on a long-term data set (16 years) and seems robust. However, parasitism rate was very scant during the first eight years included in the analysis (“a maximum of 7.4% of nests parasitized” (in [15])) and, even though authors did not provide the number of parasitized nests per year, it can be derived that only 1–3 nests were available for estimating fledging production for the period 1995–2002 [15]. Thus, it remains necessary to control for heterogeneity in data quality to allow robust conclusions [46].

Among the possible explanations of inconsistent results found in our population and the one in northern Spain, we also highlight the possibility that results may vary with ecological conditions. For example, in our study area the carrion crow is the main predator of magpie nests [11,21] and corvids have been shown to be the least sensitive to cuckoo secretions in comparison with other predators [6,41]. This suggests that cuckoo secretion would probably be less effective in magpies than in carrion crow hosts, but this fact does of course not affect the results in carrion crows. Anyhow, dependent on active predators in the different study areas and among different years, likely other results will be found.

To summarise, the fascinating possibility that hosts of the great spotted cuckoo benefit from parasitic nestlings by discouraging predators is not supported in our carrion crow or magpie population exploited by the great spotted cuckoo. Great spotted cuckoos benefit from being raised in nests without nest-mates; i.e. they grow faster and better when reared alone in nests of their main host, the magpie [19], whereas crow nestlings frequently outcompete cuckoo nestlings due to their larger size [5]. Then, the question why cuckoo nestlings should protect their carrion crow nest-mates in this situation is an important issue that remains to be answered. However, we want to emphasize that inconsistent results between our host population and the one in northern Spain [6] could be due to differences in ecological conditions between populations. Likely, the dissimilarity between the two studies may reflect a three-way interaction between environment, parasite and host species. Future research replicating this type of study in other host populations under different ecological contexts will allow to disentangle potential issues affecting the relationships between great spotted cuckoos and their corvid hosts.

## Supporting information

S1 Table Predation rate in non-parasitized versus parasitized magpie nests. (DOCX)

S2 Table Predation rate in non-parasitized versus parasitized carrion crow nests. (DOCX)
