# Supplementary material for: Great spotted cuckoo nestlings have no antipredatory effect on magpie or carrion crow host nests in southern Spain
Source: PLoS One. 2017 Apr 19;12(4):e0173080. doi: 10.1371/journal.pone.0173080 (PMC5396876; doi:10.1371/journal.pone.0173080)
Supplement: S1 Table — (DOCX) [file pone.0173080.s001.docx]

**Electronic Supplemental Information**

**Table S1.** Predation rate in not parasitized versus parasitized magpie nests in each year considering only non-manipulated nests. Results are similar when including manipulated nests.

| **Egg phase** | | | | |
| --- | --- | --- | --- | --- |
|  | **Not parasitized** | | **Parasitized** | |
|  | **%** | **N** | **%** | **N** |
| 2006 | 11.54 | 26 | 14.29 | 28 |
| 2007 | 13.33 | 60 | 9.26 | 54 |
| 2008 | 20.69 | 87 | 6.49 | 77 |
| 2009 | 28.57 | 35 | 13.51 | 37 |
| 2011 | 27.78 | 18 | 17.58 | 91 |
| 2012 | 12.28 | 57 | 12.50 | 56 |
| 2013 | 21.05 | 57 | 16.98 | 53 |
|  | |  |  |  |
| **Nestling Phase** | |  |  |  |
|  | **Not parasitized** | | **Parasitized** | |
|  | **%** | **N** | **%** | **N** |
| 2006 | 15.38 | 13 | 20.00 | 25 |
| 2007 | 24.32 | 37 | 9.52 | 21 |
| 2008 | 31.58 | 57 | 22.64 | 53 |
| 2009 | 14.29 | 14 | 14.71 | 34 |
| 2011 | 46.67 | 15 | 30.26 | 76 |
| 2012 | 14.81 | 54 | 35.42 | 48 |
| 2013 | 20.45 | 44 | 41.94 | 31 |
