# Supplementary material for: Great spotted cuckoo nestlings have no antipredatory effect on magpie or carrion crow host nests in southern Spain
Source: PLoS One. 2017 Apr 19;12(4):e0173080. doi: 10.1371/journal.pone.0173080 (PMC5396876; doi:10.1371/journal.pone.0173080)
Supplement: S2 Table — (DOCX) [file pone.0173080.s002.docx]

**Electronic Supplemental Information**

**Table S2.** Predation rate in non-parasitized versus parasitized carrion crow nests in each year considering only non-manipulated nests. Results are similar when including manipulated nests.

| **Egg phase** | | | | |
| --- | --- | --- | --- | --- |
|  | **Not parasitized** | | **Parasitized** | |
|  | **%** | **N** | **%** | **N** |
| 2006 | 12.5 | 32 | 33.30 | 3 |
| 2007 | 7.4 | 27 | 0.00 | 5 |
| 2008 | 26.3 | 19 | 23.50 | 17 |
| 2009 | 16.7 | 6 | 9.10 | 11 |
|  |  |  |  |  |
| **Nestling Phase** | | | | |
|  | **Not parasitized** | | **Parasitized** | |
|  | **%** | **N** | **%** | **N** |
| 2006 | 4.20 | 24 | 0.00 | 2 |
| 2007 | 0.00 | 9 | 0.00 | 4 |
| 2008 | 18.20 | 11 | 36.40 | 11 |
| 2009 | 0.00 | 1 | 0.00 | 4 |
